# Editorial: Neurobiological mechanisms of addiction: bridging neuroscience and clinical implications

**DOI:** 10.3389/fnhum.2026.1800164

**Published:** 2026-02-17

**Authors:** Chella Kamarajan, Ksenija Marinkovic, Ramaswamy Viswanathan

**Affiliations:** 1 Department of Psychiatry, Downstate Health Sciences University, Brooklyn, NY, United States; 2 Department of Psychology, San Diego State University, San Diego, CA, United States

**Keywords:** addiction, behavioral addiction, ERP, fMRI, fNIRS, functional connectivity, iTBS, substance use disorder

Addiction is defined as a “treatable, chronic medical disease involving complex interactions among brain circuits, genetics, the environment, and an individual's life experiences” (American Society of Addiction Medicine, 2026). Both forms of addiction, substance use disorders and behavioral addictions (e.g., gambling), involve physiological, psychological, and environmental factors that can alter the brain circuits related to reward, stress, and self-control (Koob and Volkow, 2010; Volkow and Koob, 2015; Koob and Volkow, 2016; Sussman, 2017). These neural changes may persist for a long time after a person has stopped consuming the substances or engaging in addictive behaviors (Nestler, 2001; Goldstein and Volkow, 2011; Zilverstand et al., 2018). Advancements in human neuroscience have begun to unravel the intricate neural mechanisms underlying the role of neural circuitry, neurotransmitter systems, and genetic factors in addiction, and these insights are crucial for developing effective treatment strategies. Yet, gaps remain in translating these findings into practical interventions, and addiction continues to be a formidable challenge to healthcare systems globally despite significant progress in research and clinical applications. Specifically, ongoing debates center around the best approaches to integrate neurobiological insights with clinical practices, emphasizing the need for a more comprehensive understanding of the neural processes involved in addiction and recovery. Therefore, this Research Topic aims to address the pressing need for an in-depth understanding of the neurobiological processes involved in addiction through the lens of clinical applications. By fostering a comprehensive collection of manuscripts, the Research Topic attempts to bridge the gap between neuroscientific discoveries and practical treatment applications.

The Research Topic attracted 11 articles, comprising nine original research articles, along with a mini-review and an opinion piece. Five of these articles investigated neural and cognitive mechanisms underlying alcohol use disorder (AUD). For example, an event-related potential (ERP) study by Nixon et al. examined the mid-frontal negative component (FN400) at 300–500 ms and the posterior late positive component (LPC) at 550–800 ms after stimulus presentation as neurophysiological markers in older healthy male and female drinkers aged 65–80 years while performing a working memory task. The results showed preliminary support for the hypothesis that sustained drinking among older adults may negatively impact these neurophysiological signatures of working memory. In another study, Antón-Toro et al. used magnetoencephalography (MEG) to examine binge drinking in adolescents. They reported that both the M200 (180–260 ms) and M300 (310–510 ms) components of a Go/No-Go task showed larger amplitudes at the left medial and dorsolateral prefrontal areas, supporting the hypothesis that atypical prefrontal executive engagement may underlie a vulnerability that precedes alcohol use and may contribute to the emergence of binge drinking. Further, in a novel study examining brain age using structural MRI measures of brain volume and cortical thickness, Çinaroglu et al. reported that the predicted brain age of alcohol-dependent individuals was 11.5 years greater than that of healthy controls, especially in white matter and basal ganglia structures. Additionally, an fMRI study of alcohol sensitivity by Cofresi et al. found that neural activation in dorsal anterior cingulate cortex during a Go/No-Go task was a function of intensity of alcohol craving among both low- and high-sensitivity groups, suggesting that a compensatory over-activation of fronto-cortical nodes of the fronto-cortico-striatal inhibitory control circuitry may be related to affective-motivational aspects of AUD symptomatology. Lastly, using repetitive transcranial magnetic stimulation (TMS), Durazzo et al. examined the effects of intermittent theta burst stimulation (iTBS) in the treatment of AUD and found that brain stimulation treatment led to adaptive structural and neurometabolic changes in the left dorsolateral prefrontal cortex, which was the site of stimulation.

Some of the studies examined other substances. Yan et al. investigated whether decision-making deficits may have predated drug abuse due to potential familial susceptibilities or emerged as a consequence of chronic drug use by comparing decision-making styles between heroin-dependent individuals and their siblings using behavioral measures of task performance and questionnaires. Results showed that deficits in trait-like and risk-taking-related decision-making styles were shared by abstinent heroin-dependent individuals and their unaffected siblings, suggesting familial vulnerabilities implicated in the development of heroin dependence. Further, a study by Kulaksızoğlu et al. investigated the levels of several neural and other biological parameters, i.e., serum zonulin, brain-derived neurotrophic factor (BDNF), total oxidant status (TOS), total antioxidant status (TAS), and oxidative stress index (OSI), in individuals who used A4-size papers impregnated with synthetic cannabinoids (A4 use), a novel form of substance use. The findings suggested that individuals with A4 use showed impaired neuroplasticity, along with disrupted oxidative stress balance, evidenced by BDNF, TOS, and OSI levels, suggesting alterations in multiple neurobiological measures due to A4 use.

Two additional articles examined behavioral addictions such as internet gaming disorder and internet pornography addiction. Using resting-state fMRI, Zhao et al. examined dynamic functional connectivity in adolescents with internet gaming disorder and found aberrant connectivity within the amygdala-hippocampal complex and its interaction with the visual network. In another study, Shu et al. examined the impact of internet pornography addiction on brain function using functional near-infrared spectroscopy (fNIRS). Results showed that the group that frequently viewed pornographic videos, compared to the group that viewed pornography less frequently, exhibited altered functional connectivity in several brain regions, along with hyperactive parasympathetic activity and more pronounced sexual arousal, revealing extensive involvement of both central and autonomic nervous systems in pornographic addiction.

Additionally, integrating both theoretical and empirical work on developmental neuroscience, attachment theory, and psychodynamic psychotherapy, the review article by Unterrainer has attempted to reconceptualize addiction as a disorder rooted in disrupted attachment and altered brain function. Drawing on both clinical and research findings, this article explores how early relational trauma contributes to dysregulation of stress-response systems and functional changes in brain regions underlying self-awareness, emotion regulation, and reward processing. This work thus supports a view of addiction as a disconnection from bodily and relational signals associated with early attachment experiences, and it adheres to a more integrative, developmentally informed treatment model. Lastly, the opinion article by López-Caneda and Almeida-Antunes is an attempt to gather evidence for the theory that manifestation and persistence of addictive behaviors are due to a lack of ability to suppress drug-related memories that contain aspects relating to the “wanting system” (the automatic motivational pull toward drug-seeking behaviors). The article proposes that individuals who are addicted to substances and those who are prone to addiction are unable to suppress episodic drug-related memory, along with the beliefs and semantic associations it evokes. It further theorizes that this impairment in memory suppression may contribute to the maintenance of incentive salience of drug-associated stimuli, thereby potentiating or perpetuating addictive behaviors by maintaining maladaptive motivational responses associated with drug addiction.

In sum, each article in this Research Topic collection has made a significant contribution to further improving our scientific understanding of addiction, with the potential to inform clinical applications. We hope that the findings revealed by this collection of articles, as part of the broader efforts of the scientific and clinical communities around the world, will contribute toward the development of better methods and techniques for preventing, diagnosing, and treating substance use disorders (SUD) as well as behavioral addictions.
